# Plaque morphology detected with Duplex ultrasound before carotid angioplasty and stenting (CAS) is not a predictor of carotid artery in-stent restenosis, a case control study

**DOI:** 10.1186/1471-2377-13-163

**Published:** 2013-11-05

**Authors:** Katrin Wasser, André Karch, Sonja Gröschel, Janin Witzenhausen, Klaus Gröschel, Mathias Bähr, Jan Liman

**Affiliations:** 1 Department of Neurology, University of Göttingen, Robert-Koch-Str. 40, 37075 Göttingen, Germany; 2 Department of Psychiatry, University of Mainz, Untere Zahlbacher Str. 8, 55131 Mainz, Germany; 3 Department of Neurology, University of Mainz, Langenbeckstr. 1, 55131 Mainz, Germany

**Keywords:** Carotid artery stenosis, Stent, Angioplasty, Restenosis, Plaque, Duplex ultrasound

## Abstract

**Background:**

In-stent restenosis (ISR) is an important factor endangering the long-term safety and efficacy of carotid artery angioplasty and stenting (CAS). It is plausible that soft vulnerable plaques are more likely to be injured during CAS procedure and are therefore more likely to initiate the cascade finally leading to ISR. The aim of this study was to investigate if plaque morphology detected by a simple applicable Duplex ultrasound score before CAS can be used as a predictor for ISR.

**Methods:**

Within a prospectively collected single-centre CAS database of 281 patients (comprising 300 arteries) with high-grade carotid artery stenosis, who underwent CAS between May 2003 and January 2013, we conducted a nested case–control study. Plaque morphology before CAS was analysed by a blinded investigator and each parameter of the Total Plaque Risk Score (TPRS) as well as the whole score was evaluated with regard to its diagnostic validity for ISR.

**Results:**

We analysed the data of 10 patients with ISR and 50 patients without ISR. There were no significant differences with respect to baseline characteristics, vascular risk factors, and degree of stenosis between patients with and without ISR. The duration of follow-up was longer in patients with ISR (p = 0.024) and these patients were more likely to show increased PSV (p = 0.012) immediately after CAS than patients without ISR. Neither individual parameters of the TPRS score nor the score as a whole were suitable as a diagnostic test for ISR development.

**Conclusions:**

In the present study we could demonstrate that the non-contrast enhanced DUS of the pre-interventional plaque formation cannot be used as a predictor for the development of ISR. Evaluating a more sophisticated, but not routinely available approach e.g. by ultrasound based plaque perfusion imaging or CT based plaque analysis could be helpful in the future in order to assess the role of plaque morphology in the context of ISR development.

## Background

Atherosclerotic stenosis of the internal carotid artery (ICA) is known as a major risk factor for disabling stroke and death. Carotid endarterectomy (CEA) in combination with best medical treatment of concomitant cerebrovascular risk factors is currently the therapy standard for patients with symptomatic ICA stenosis and some patients with high-grade asymptomatic ICA stenosis. Nevertheless, carotid angioplasty and stenting (CAS) has been used as the treatment of first choice in many centres, despite the fact that randomized controlled trials and subsequent meta-analyses could not provide evidence for a general superiority of CAS over CEA [1-6]. However, the results of these trials have been interpreted very controversially resulting in conflicting recommendations in various current guidelines [7,8]. Although CEA is still the goldstandard therapy for most patients, there is accumulating evidence that a subgroup of patients aged <70 years may benefit from a CAS intervention [5,9-11]. One factor that could influence the long-term safety and efficacy of CAS is an in-stent restenosis (ISR); indeed, we could recently show that the combined stroke and death rate during long-term follow-up was significantly higher in the group of patients suffering from ISR compared with patients without ISR [12]. Therefore, it is of highest interest to identify predictors of carotid artery in-stent restenosis in CAS-treated patients.

A possible predictor of ISR could be the lesion characteristics of the stenotic artery. Up to now it could be shown that (regardless of a CAS intervention) a compound score of plaque surface irregularity, echoluency and texture characteristics can predict the risk of stroke [13]. Furthermore, new ischemic lesions as detected with MRI after CAS are closely related to the plaque vulnerability. It could also be shown that fibrolipid plaques are associated with a higher burden of new ischemic lesions [14,15].

Taking pathophysiological mechanisms of ISR development into account, it seems plausible that plaque morphology is not only a predictor of ischemic events during CAS, but may also be associated with a higher risk of the development of an ISR. We know from coronary artery angioplasty and stenting that vascular injury, which is caused by balloon inflation and stent placement, leads to inflammatory processes, which, themselves, play the pivotal role in the pathogenesis of ISR, finally causing neointimal proliferation through the stent meshes [16-18]. In the context of CAS Petric et al. demonstrated that calcified plaques bear a lower risk of arterial injury although they could be exposed to higher dilation pressure while CAS. The authors concluded that this might reduce the initial stimulus for ISR [19].

The Total Plaque Risk Score (TPRS) described by Prati et al. [13] has proven its value in the prediction of future strokes in asymptomatic patients and considers 1) the degree of the stenosis, 2) echogenicity, 3) texture, and 4) surface characteristics. We used this score, which can easily be evaluated with the commonly available, cost-effective and non-invasive Duplex ultrasound investigation, in order to investigate the influence of plaque morphology on ISR development.

## Methods

### Patients

The design of the core study has been published in detail, recently [12]. Within a prospectively created single-centre CAS database of 281 patients (comprising 300 arteries) we conducted a nested case–control study. All patients suffered from a symptomatic carotid artery stenosis ≥70% or an asymptomatic carotid artery stenosis ≥90% (degree of stenosis was measured according to the European guidelines (ECST) [20]) and underwent CAS between May 2003 and January 2013. For inclusion in this study, only patients with a complete and well-documented pre-interventional Duplex ultrasound were considered. A total of 14 patients (4.7%) developed ISR during long-term follow-up within our database. Of these 14 patients, 10 (71.4%) had well analysable, pre-interventional Duplex ultrasound image. Furthermore, a control-group of 50 patients (23.8%) without ISR during long-term follow-up was randomly chosen from those meeting above named inclusion criteria for cases (n = 210). All cardiovascular risk factors and clinical outcome parameters were recorded by experienced stroke neurologists (K.G. and K.W.).

The current study has been conducted in accordance with International Conference on Harmonisation/Good Clinical Practice (ICH/GCP) guidelines and was approved by the local Ethics committee of the University Hospital Göttingen, Germany.

### Doppler and duplex sonography

The diagnosis of carotid artery stenosis and ISR was made by carotid duplex ultrasound imaging using a combination of direct and indirect criteria, which have been described in detail, recently [12,21]. Peak systolic flow velocities (PSV) within the stenosis and post-stenotic internal carotid artery, end diastolic flow velocity in the stenosis, internal carotid artery/common carotid artery PSV ratio, and pre-stenotic and post-stenotic frequency patterns were determined as direct criteria for the local degree of stenosis. Flow characteristics of the supra-trochlear artery and the anterior cerebral artery as well as the pulsatility of the ipsilateral common carotid artery were taken into account as indirect criteria for a high-grade stenosis. The degree of carotid stenosis at baseline was graded according to angle corrected maximum intrastenotic peak systolic velocities according to ECST criteria as follows: baseline stenosis ≥70% = PSV ≥200 cm/s, baseline stenosis ≥80% = PSV ≥300 cm/s, baseline stenosis ≥90% = PSV ≥400 cm/s.

As there is a lack of valid ultrasound criteria for the definition of an ISR and as the current literature supposes different criteria, [5,6,22] we used locally adopted criteria with a PSV ≥300 cm/s as a key feature representing an ISR of ≥70%, as this velocity is best evaluated in the literature and consistent with our CT or angiography based control examinations of ISR detected by DUS [12,21].

**Figure 1:**
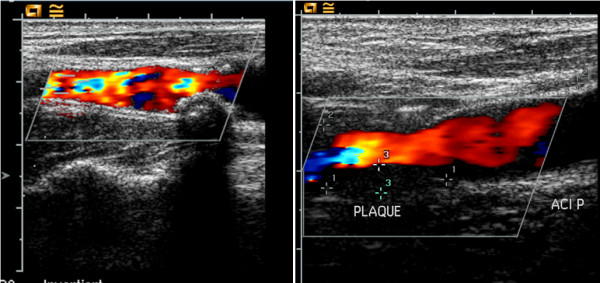
**Shown are two plaque types with different plaque scores.** The left plaque would be classified as stenosis < 40%; intermediate echogenicity; heterogenous texture and irregular surface. Plaque score: 4; reversed score: 4. The right plaque would be classified as stenosis <40%, low echogenicity, homogeneous texture and smooth surface. Plaque score: 1, reversed score: 4.

Regarding to plaque morphology we collected data with respect to four different plaque qualities as described in detail by Prati et al. [13] (Table 1). The first parameter is the degree of stenosis. According to Prati et al., patients with a stenosis >40% using NASCET criteria were scored with “1”. All of our patients fulfilled that criterion. Secondly, the echogenicity was graded from 1 to 3 according to the Gray-Weale modified score [23-25]. Thirdly, the texture was graded “1” if a heterogeneous echo pattern was detected and “0” if a homogeneous echo pattern was present. Fourthly, the surface characteristic was graded “0” if the plaque contour was smooth or “1” if it was irregular. The TPRS was computed by adding the values of the four parameters and could range from 0 to 6 as summarized in Table 1. We also composed an alternative score based on the latter three parameters with inversed values for the parameter “echogenicity”, because an anechoic plaque is as well as a heterogeneous texture and an irregular surface considered being associated with a higher vulnerability of the plaque (Figure 1).

**Table 1:** The Total Plaque Risk Score (TPRS)

**Parameter**		**Score = 0**	**Score = 1**	**Score = 2**	**Score = 3**
I)	Degree of stenosis	< 40% (NASCET)	≥ 40% (NASCET)		
II)	Echogenicity		Low echogenicity or echolucency	Intermediate echogenicity	Hyper-echogenicity
III)	Texture	Homogeneous	Heterogeneous		
IV)	Surface	Smooth	Irregular		

All examinations were performed according to a standardized protocol in the same vascular laboratory with the same ultrasound equipment (Acuson Sequoia™ 512, Siemens, San José, CA) under the supervision of an experienced, board certified vascular neurologist (K.G.).

Patient data sets were collected by a second person of the study group (K.W.) and subsequently analysed by an experienced, board certified vascular neurologist, blinded to outcome parameters (J.L.).

### CAS procedure

CAS was carried out by experienced interventional neuroradiologists under anaesthesiological stand-by using a transfemoral approach. Stent-type and the use of filter-based neuroprotection devices were chosen at the discretion of the interventionalists. Only patients scheduled for elective CAS were recorded, patients in unstable clinical conditions or with stroke in evolution were excluded. All patients received orally administered acetylsalicylic acid (100 mg/d) and clopidogrel (75 mg/d) at least 3 days before the procedure or if that was not possible due to CAS procedure earlier than 3 days after admission they received a loading dose of 600 mg clopidogrel and 300 mg of acetylsalicylic acid. Clopidogrel was continued for 6 to 12 weeks after CAS in a daily dosage of 75 mg and aspirin was administered life-long in a dosage of 100 mg/d. After being routinely monitored in our intensive care or stroke unit overnight for at least one day all patients could be discharged to normal ward or home.

### Follow-up protocol

All patients were seen for serial duplex sonography and clinical follow-up at the hospital’s outpatient clinic at 3, 6, and 12 months after the CAS-procedure and every 6 months thereafter.

### Statistical analysis

Categorical variables were expressed as count and percentages, continuous values as mean ± standard deviation (SD), or as median values with the corresponding interquartile range (IQR) as appropriate. For univariate comparisons of categorical data, Chi-square tests with Yates’ correction and Fisher’s exact test were used as appropriate. For univariate comparisons of continuous outcome variables, two-sided t-tests and Wilcoxon-Ranksum tests were applied dependent on distribution features and variances of the outcome variable.

TPRS was evaluated as a diagnostic test by using receiver-operating characteristic (ROC) curves. Sensitivity and specificity was calculated for different cut-off values to establish the best cut-off according to the Youden-index. Predictive values were calculated with the Bayes formula using prevalence estimates from the initial cohort study.

In an exploratory secondary analysis, respective score items were assessed individually as potential diagnostic tests for predicting in-stent restenosis in patients who underwent CAS.

All statistical analyses were performed with Stata 11 (StataCorp, College Station, Texas).

## Results

A total of sixty patients (48 men and 12 women) treated with CAS were analyzed in this study including 10 cases (patients with ISR) and 50 controls without ISR. Cases and controls showed no significant differences with respect to baseline characteristics, vascular risk factors, and degree of stenosis (Table 2). ISR cases were followed up for longer (p = 0.024) and were more likely to show increased PSV (p = 0.012) post intervention than controls.

**Table 2:** Baseline characteristics of study population

**Variable**	**Data**		
	**ISR ≥70%**	**No ISR**	**p value**
N	10	50	
Age, years	69.8 ± 7.6	68.2 ± 8.7	0.580
Female sex	4 (40%)	8 (16%)	0.101
Right side	6 (60%)	19 (38%)	0.294
Symptomatic carotid stenosis	4 (40%)	32 (64%)	0.178
Stroke	2 (20%)	21 (42%)	0.291
Hemispherical TIA	2 (20%)	10 (20%)	1.000
Arterial Hypertension	10 (100%)	48 (96%)	1.000
Hyperlipidemia	10 (100%)	36 (72%)	0.098
Tobacco use	4 (40.0%)	13 (26%)	0.448
Diabetes mellitus	2 (20%)	11 (22%)	1.000
Coronary artery disease	3 (30%)	16 (32%)	1.000
Peripheral occlusive arterial disease	3 (30%)	7 (14%)	0.347
Atrial fibrillation	1 (12.5%)	3 (6%)	0.528
CEA restenosis	3 (30%)	6 (12%)	0.163
Contralateral ICA occlusion	3 (30%)	7 (14%)	0.347
Contralateral ICA stenosis ≥70%	2 (20%)	13 (26%)	1.000
Stenosis ≥ 90% before CAS	7 (70%)	22 (44%)	0.175
Median follow-up time (month, IQR)	15 (4.7 - 35.4)	40.2 (26.7 – 59.3)	0.024*
PSV >120 cm/s after CAS	4 (40%)	3 (6%)	0.012
Re-interventions	6 (60%)	0 (0%)	<0.001

*significant difference.

### Evaluation of plaque morphology

When investigating plaque morphology before CAS all patients in the ISR group as well as in the control group met the criteria of stenosis degree >40%.

Echogenicity was scored as described by Prati et al. [13]. In the ISR group 50% (5 of 10 patients) met the criteria for score 1, 30% (3 of 10 patients) for score 2 and 20% (2 of 10 patients) for score 3. The mean score in the ISR group was 1,7. In comparison, in the control group 34% scored 1 (17 of 50 patients), 54% scored 2 (27 of 50 patients) and 12% scored 3 (6 of 50 patients) with a mean echogenicity score of 1.78. No significant difference was detectable between the two groups (p = 0,46).

As the degree of hyperechogenicity correlates with the calcification and might also be beneficial, in contrast to a “soft plaque”, we also reversed the TPRS scoring system in this point scoring 1 for hyperechogenicity and 3 for low echogenicity. This sub score showed a hyperechogenicity in 2 of 10 patients (20%) in the ISR group and in 6 of 50 patients (12%) in the group of patients without ISR and a low echogenicity in 5 of patients with ISR (50%) and in 17 of 50 patients without ISR (34%). The mean of the reversed echogenicity score was 3,8 in the ISR group and 3,44 in the group without ISR (p = 0,46).

Texture was described as homogenous (score 0) or heterogeneous (score 1). In the ISR group 40% (4/10 patients) scored 0 and 60% (6/10 patients) scored 1, whereas 46% (23/50 patients) scored 0 and 54% (27/50 patients) scored 1 in the control group. Again no significant difference between the two groups was detectable (p = 0,53).

The surface was described as either smooth (score 0) or irregular (score 1). In the ISR group 10% (1/10 patients) scored 0 and 90% (9/10 patients) scored 1, whereas 34% (17/50 patients) scored 0 and 66% (33/50 patients) scored 1 in the control group. Again no significant difference between the two groups was detectable (p = 0,62).

In the next step, we performed a ROC analysis for TPRS as well as for all individual parameters and calculated sensitivity and specificity, as well as negative predictive value and positive predictive value for each parameter (Table 3). Neither the TPRS score nor one of the individual parameters showed an acceptable validity so that it could be used as a predictive diagnostic test for ISR in patients undergoing CAS.

**Table 3:** Diagnostic validity and statistical data

	**TPRS**	**Inverse TPRS**	**Echo-genicity**	**Texture**	**Surface**
AUC (95%CI)	0.54	0.64	0.46	0.53	0.62
	(0.36-0.72)	(0.45-0.83)	(0.25-0.67)	(0.36-0.70)	(0.50-0.74)
Cut-off	≥ 3	≥ 4	≥ 3	≥ 1	≥ 1
Sensitivity	0.70	0.80	0.20	0.60	0.90
Specificity	0.40	0.42	0.88	0.46	0.34
PPV*	0.06	0.08	0.09	0.06	0.07
PNV*	0.96	0.97	0.95	0.95	0.98

*Positive and negative predictive value given the ISR prevalence of 0.06 in the overall population of CAS-treated individuals of this study.

## Discussion

CAS might be the therapy of choice for patients younger than 70 years [5,9-11]. The on average young age of CAS patients highlights the importance of a good long-term clinical outcome and the need for potentially predictive factors concerning the development of an ISR. Although different approaches, especially regarding laboratory parameters, have been made no reliable parameter is available to date which can sufficiently predict an increased risk of ISR [14,21,26,27].

Duplex sonography (DUS) has been proven over years to be able to assess carotid plaque morphology with regards to plaque surface and the plaque structure. In 2004 Willfort-Ehringer and co-workers applied DUS to assess the influence of pre- interventional plaque morphology on Stent expansion in a 2 years follow up study [28]. They evaluated the plaque morphology concerning the echoluency of the preinterventional plaque and discriminated between 7 different plaque types ranging from soft to very hard with extensive shadowing. They found that an increased calcification of the pre-interventional plaque composition is associated with a decrease of stent expansion [28] which is known as a risk factor for the development of ISR [12,22].

Bearing these studies in mind we sought to study the influence of pre-interventional plaque morphology on ISR by an easy to use and reliable plaque scoring system.

Intima injury is supposed to be the initial trigger of ISR. Petric et al. showed that the risk of intima injury was lower in calcified plaques in comparison to soft plaques strengthening the hypothesis of the “vulnerable” soft plaque [19]. Therefore, it could be possible that a soft plaque may be associated with a higher risk of the development of an ISR.

We retrospectively analyzed the ultrasound data of patients who underwent CAS in our department between 2003 and 2012 and analyzed the plaque morphology regarding to the TPR score published by Prati et al. [13]. Interestingly we were not able to detect any influence of either scoring parameters on the occurrence of ISR in the course of the evaluation. But, as described by Willfort-Ehringer and us earlier, we were also able to correlate the echogenicity of the preinterventional plaque with the occurrence of incomplete stenosis dilatation, which is, as we were able to show earlier and again an independent indicator for ISR occurrence [12,28].

One main shortcoming of our study, besides the retrospective design, is the small sample size. We were only able to analyze the data of 10 patients suffering from an ISR and 50 controls, leaving this study underpowered for the detection of small differences within the analyzed factors. However, the results of this study provide evidence that no plaque parameter within the TPRS is able to predict ISR in CAS patients with acceptable validity.

## Conclusion

In the present study we sought to evaluate a standard, easy to use, broadly applicable and reliable system. It might well be, that the non-contrast enhanced DUS of the pre-interventional plaque formation is not sufficient enough and it would be more advisable to investigate a more sophisticated approach either by ultrasound based plaque perfusion imaging, or CT based plaque analysis, lacking the immediate broad applicability. In summary, the pre-interventional assessment of plaque morphology with conventional DUS by using the TPRS is not useful in order to distinguish between patients who are likely to suffer from ISR. Further studies will need to analyze prospectively the usefulness of e.g. Plaque perfusion techniques and DUS or CT techniques in order to reevaluate pre-interventional plaques as possible predictive markers for the development of ISR.

## Competing interests

The authors declare that they have no competing interests.

## Authors’ contributions

KW Searched the database, selected the patients, participated in statistical analysis, prepared the manuscript draft and participated in the study design. AK performed the statistical analysis and helped to draft the manuscript. SG participated in creating the database and helped drafting the manuscript. JW participated in creating the database and helped drafting the manuscript. KG participated in creating the database and helped drafting the manuscript. MB participated in study design and outline and helped to draft the manuscript. JL designed the study, participated in drafting the manuscript, did the figure work and analysed the ultrasound data. All authors read and approved the final manuscript.

## Pre-publication history

The pre-publication history for this paper can be accessed here:

http://www.biomedcentral.com/1471-2377/13/163/prepub
